# Time for a Change: Personal Experiences With COVID-19 and Diabetes

**DOI:** 10.1177/1932296820928083

**Published:** 2020-05-22

**Authors:** Frank Best

**Affiliations:** 1 Die Diabetes-Praxis, Essen, Germany

**Keywords:** COVID-19, diabetes, management, personal experience

In memoriam Dr Li Wenliang

The first patient with COVID-19 in Germany was diagnosed on January 27th. Until today (mid-April) we count 137 000 infected and about 4000 deaths.

COVID-19 has changed our daily life considerably.

I am looking after about 1500 patients, all of them with diabetes, 40% of them with diabetes type 1. About half of my patients are beyond 60 years of age, male, obese, and/or on antihypertensive drugs.

Early in March we decided to cut down personal contacts to patients to a minimum. We have switched from quarterly dates to video sessions. Diabetes education too.

I have learned, . . .

. . . that it is very important to let your patients know, you are within reach! That they can contact you every time by phone or email. That they can come to the office, if there is no other possibility, for example, with a diabetic foot. That special situations require a different approach, but that you will never leave them alone!

. . . that a challenge like COVID-19 can be helpful. Most of the folks in their 70s or 80s are happy using continuous glucose monitor (CGM), but they wouldn’t upload their data to a web portal. Now they have to (with the help of the family). And we can share screens and data on a video session, as if they were in the practice. That makes them proud and gives confidence.

. . . that many are scared by this unknown threat. Asking: “How are you dealing with it?”. Knowing that I am type 1 for some 50 years. “I take care!”—And we can discuss frankly the risks and the strategies to reduce them.

. . . that theoretically there is a technical solution for my problems: talking to patients (nearly face to face), sharing screens, and looking at data.

My home town is among the 10 biggest cities in Germany. But there is no broadband internet connectivity in every region. So, video conferences break up very often. The accredited video software does not work with every internet browser—because of security reasons. You have to explain to the patient how to get and install the correct browser.

Companies like Medtronic, Dexcom, and Abbott have their own web portals, where patients can upload their CGM data and share them with their diabetes team. The medtronic (MDT) server breaks down regularly (“An error has occurred. Try again later.”). Abbott updates their LibreView portal without notice, cutting off their customers for hours. The hotlines are always busy.

Meter companies like Roche require patients to use special interfaces to upload their blood glucose values. I have such an interface, most patients have not!

I have learned that I could have known about this pandemic and that I could have been prepared better. Bill Gates delivered an impressive TED talk in 2015 (“The Next Outbreak”).^1^ I missed it.

In October 2019, the Johns Hopkins Center for Health Security hosted a pandemic tabletop exercise called *Event 201*,^2^ describing in detail, what we are facing today. I missed it.

In the future I predict, . . .

. . . that it will be our task as doctors to support patients in their approach, that the data belong to them. Not to Abbott, Dexcom, Medtronic, or Roche.

. . . that proprietary solutions are unnecessary obstacles. I think the OpenSource idea and a new kind of “community mentality” will gain more and more influence. It was impressive to see that in 48 hours during the hackathon “WirVsVirus”^3^ over 40 000 people discussed and developed projects for helping others. It is impressive to see that while governments are discussing, if and which app might be suitable for contact tracking, the community is presenting such an app in a ß-version (CoEpi).^4^

. . . that globalization has reached a tipping point. The credo of our economy (“more, cheaper”) led to a production where wages are low, led to “optimization” (closing hospitals, putting off health workers), and led to producing pharmaceuticals in developing countries at an unbelievable price. We are suffering from that mentality now. We need to accept that quality has a price and that vital products have to be produced nearby.

. . . that this pandemic will have a deep influence on the circumstances and the mentality that will develop in our communities. Solidarity vs egoism. Dr Roberto Burioni, virologist in Milano, said in an interview: “We are sharing the same history and culture. And as well common threats. Perhaps, when this is over, we will be a united Europe. This is a pleasant thought.”^5^

Dr Li Wenliang^6^ in Wuhan was silenced by government authorities, when he rose his voice to warn colleagues and countrymen about COVID-19. A short time later he succumbed to the disease.

He has my deepest respect.
